# Supplementary material for: Prognostic Profiling of the EMT-Associated and Immunity-Related LncRNAs in Lung Squamous Cell Carcinomas
Source: Cells. 2022 Sep 15;11(18):2881. doi: 10.3390/cells11182881 (PMC9497331; doi:10.3390/cells11182881)
Supplement: Supplementary file 1 [file cells-11-02881-s001.zip › information of supplemental metarial.pdf]

Figure S1

Combine the four data sets and remove Batch Effect GEO database.

Figure S2

Expression of most immune checkpoints comparing the two subtypes both in TGCA and GEO database.

Figure S3

Distribution of uniformly upregulated TF in GEO subtypes.

Ns; $p > 0.05$; **** $p < 0.0001$.

Table S1 Clinical data screening

Table S2 TCGA.cormat.EMT.lncrna

Table S3 GEO.cormat.EMT.lncrna

Table S4 TCGA_LUSC_subtype

Table S5 GEO_LUSC_subtype

Table S6 TCGA.mut.dat

Table S7 TCGA.TF_activities.anova.res

Table S8 GEO.TF_activities.anova.res

Table S9 ALL.TF.degs.sig.tet

Table S10 ALL.TF.targets.enrich.kegg
