# Supplementary figures and images for: Prognostic Profiling of the EMT-Associated and Immunity-Related LncRNAs in Lung Squamous Cell Carcinomas
Source: Cells. 2022 Sep 15;11(18):2881. doi: 10.3390/cells11182881 (PMC9497331; doi:10.3390/cells11182881)

category C1 C2

**A**

TCGA

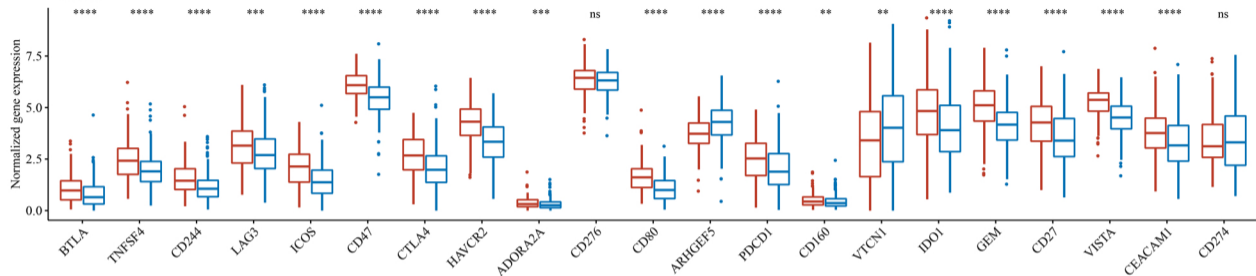**B**

GEO

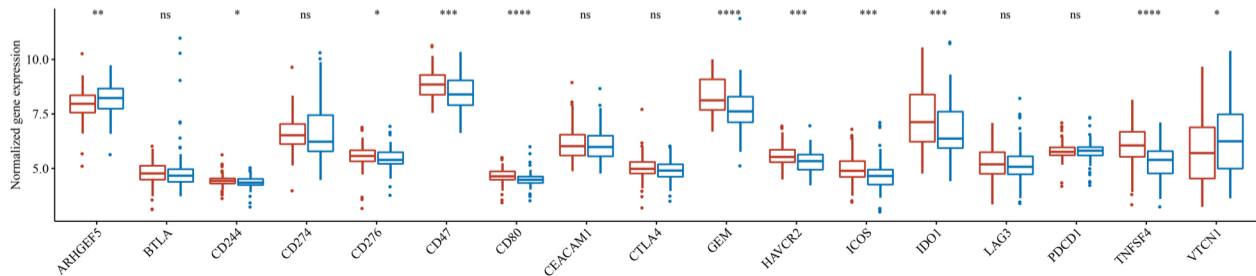

Supplement: Supplementary file 1 [file cells-11-02881-s001.zip › Fig.S1.pdf]

## Cluster

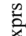

Supplement: Supplementary file 1 [file cells-11-02881-s001.zip › Fig.S2.pdf]

**A**

—●— GSE29013    —●— GSE30219  
—●— GSE37745    —●— GSE50081

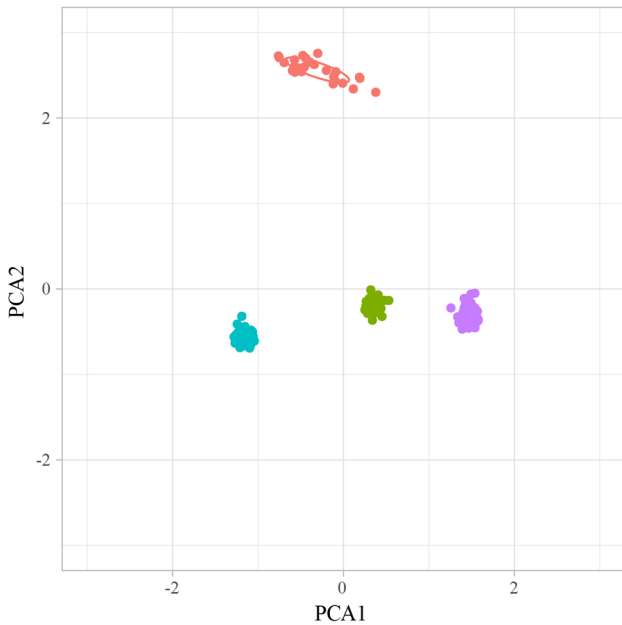**B**

—●— GSE29013    —●— GSE30219  
—●— GSE37745    —●— GSE50081

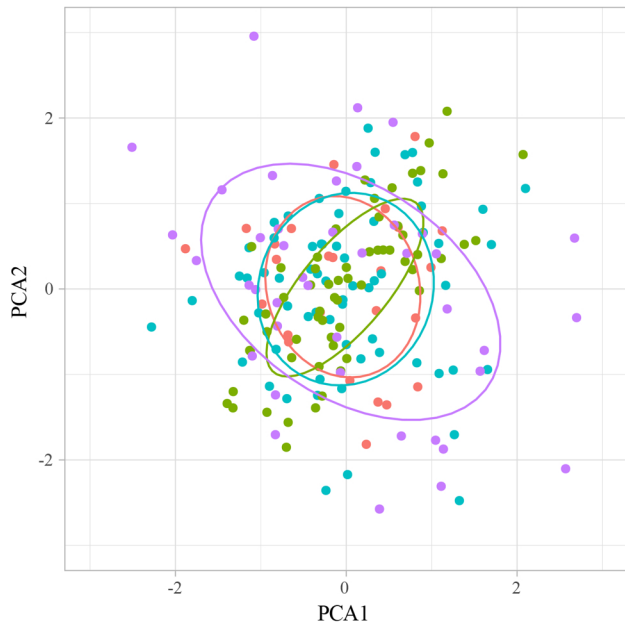

Supplement: Supplementary file 1 [file cells-11-02881-s001.zip › Fig.S3.pdf]
